# Teddy and I Get a Check-Up: A Pilot Educational Intervention Teaching Children Coping Strategies for Managing Procedure-Related Pain and Fear

**DOI:** 10.1155/2016/4383967

**Published:** 2016-04-07

**Authors:** Jessica S. Dalley, C. Meghan McMurtry

**Affiliations:** ^1^Department of Psychology, University of Guelph, 50 Stone Road East, Guelph, ON, Canada N1G 2W1; ^2^Children's Health Research Institute, 800 Commissioners Road East, London, ON, Canada N6C 2V5; ^3^Department of Paediatrics, Schulich School of Medicine & Dentistry, Western University, 800 Commissioners Road East, London, ON, Canada N6C 2V5

## Abstract

*Background*. Pediatric medical information provision literature focuses on hospitalization and surgical procedures, but children would also benefit from an educational program regarding more commonly experienced medical procedures (e.g., needles, general check-up).* Objective*. To determine whether an evidence-based educational program reduced children's ratings of fear of and expected pain from medical stimuli and increased their knowledge of procedural coping strategies.* Methods*. An educational, interactive, developmentally appropriate Teddy Bear Clinic Tour was developed and delivered at a veterinary clinic. During this tour, 71 5–10-year-old children (M_age_ = 6.62 years, SD = 1.19) were taught about medical equipment, procedures, and coping strategies through modelling and rehearsal. In a single-group, pretest posttest design, participants reported their fear of and expected pain from medical and nonmedical stimuli. Children were also asked to report strategies they would use to cope with procedural fear.* Results*. Children's ratings for expected pain during a needle procedure were reduced following the intervention. No significant change occurred in children's fear of needles. Children reported more intervention-taught coping strategies at Time 2.* Conclusions*. The results of this study suggest that an evidence-based, interactive educational program can reduce young children's expectations of needle pain and may help teach them procedural coping strategies.

## 1. Introduction

Many children experience distress when visiting a doctor or undergoing needle procedures. Children are fearful of experiencing pain during medical procedures and may associate going to the doctor's office with needle procedures [1, 2] (fear is defined as “an immediate alarm reaction to danger,” which triggers escape behaviour and an intense physiological response [3, 4]). Children's medical experiences can have both short- and long-term consequences on their mental and physical health [4–8]. For example, when the fear and pain associated with needle procedures are not addressed, individuals may be at long-term risk for experiencing increased procedure-related pain, developing needle phobia, and not adhering to vaccination recommendations [4, 7, 9]. There is general consensus that providing accurate medical information may result in more positive emotional and physical outcomes for children [5]. Children may benefit from an evidence-based educational program on general medical procedures that occur quite commonly throughout childhood (e.g., vaccinations, check-ups during well-child visits).

The content of such an educational program for children should include (1) procedural information (e.g., how long the procedure will take, who will participate, what tools will be used, and why the procedure is necessary) [5, 6]; (2) sensory-based information (e.g., physical and emotional sensations children may experience during the procedure, including pain) [6, 10]; and (3) coping strategies such as distraction and deep breathing exercises [11–13]. A meta-analysis of information provision research supported the “dual preparation hypothesis,” which states that providing a combination of sensory- and procedure-based information is more effective than either alone [10]. It is essential to ensure that children have realistic expectations of what they will experience in terms of pain as children who underestimate the amount of pain they will experience in a medical procedure may subsequently overpredict the pain they will experience in later procedures [14]. In terms of delivery, information must be provided using language appropriate for the child's level of cognitive functioning [6] with an emphasis on using literal and concrete terms [5]. The educational process should be interactive including modeling, demonstration, and rehearsal of new concepts and skills [5].

Well-child visits, vaccinations, and outpatient care occur commonly throughout childhood and may therefore play a strong role in the development of children's medical fears. Thus, children would seemingly benefit from a broad-scope educational program about general medical procedures [8]. However, most information provision research is in the context of hospitalization and/or surgical procedures [5, 6, 15–17], and research regarding how to effectively prepare children for more needles and other common minor medical procedures is needed [8]. Furthermore, most published reports of an educational tour in any medical setting do not include an evaluation of the tour's effects using measures with established psychometric properties or sufficient descriptions of the program [16–19]. Existing studies with an evaluative component have been specific to hospitalization, surgical procedures, or vaccinations and did not include pain as an outcome measure [16–21]. Thus, an evaluation of a clearly described educational program with fear and expected pain outcome measures is needed, followed by research adapting the program to maximize clinical feasibility.

The current pilot study tested an interactive, developmentally appropriate “Teddy Bear Clinic Tour” program combining the suggested strategies of medical information provision research. Children attended a tour of a primary care veterinary clinic with their own stuffed animal that received a “check-up.” While this intervention was designed to provide information about a general medical procedures for children, the veterinary clinic was a relevant setting as many of the same tools are used at a veterinary clinic (stethoscope, otoscope, needles, etc.) and the patient rooms are quite similar (e.g., examining table). Due to these similarities, the information provided to children was described in the context of both a pet's visit to the veterinarian as well as a child's visit to primary healthcare centres. A single-group pretest posttest design was used to test the following hypotheses: children would report decreased ratings of fear of (H_1_) and expected pain (H_2_) from medical stimuli, as well as an increased number of coping strategies (H_3_) following the intervention.

## 2. Methodology

### 2.1. Participants

A convenience sample was recruited from groups of children already scheduled to attend a Teddy Bear Clinic Tour at the Hill's Pet Nutrition Primary Healthcare Centre of the Ontario Veterinary College (OVC). Seventy-five children participated in a Teddy Bear Clinic Tour, and 71 children participated in the research study, for a 95% recruitment rate. The sample was 86% female (*n* = 61) and 14% male (*n* = 10). The participants ranged from 5 to 10 years old (M_age_ = 6.62, SD = 1.19), with the majority (78%) of participants being 5–7 years old. A sample of 71 was sufficient to detect a medium effect size at power = .80, *α* = 0.05 in the main analyses [22, 23].

### 2.2. Educational Program: Teddy Bear Clinic Tour

During a highly structured, 45-minute tour children spent most of their time learning about general medical equipment in the main exam room (e.g., needles, stethoscope, and otoscope); they also briefly visited X-ray machines in the radiology room and physical therapy in the rehabilitation room. Children were taught coping strategies throughout the entire tour, including an initial introduction to the coping strategies at the beginning of the tour, followed by reminders and further rehearsal during each scheduled room of the tour.

The tour script (see the Appendix) was based on the pediatric medical communication literature. The script utilized concrete language and short statements regarding the reason for medical procedures, the medical equipment used, and the sensations children would feel, with analogies included when applicable (e.g., using a stethoscope makes listening to your breath easier because it is louder, like turning up the volume on a TV). There is some consensus that children learn information best through demonstration [24] and therefore medical procedures were described and demonstrated by tour leaders on each child's stuffed animal. Based on procedural coping literature, coping strategies taught to children included the use of verbal, cognitive, and physical distraction, including humour, deep breathing, and holding their stuffed animal [2, 12, 13, 25]. The tour was piloted without data collection using two groups of children aged 5–12 (for a total of approximately 25 children) and was adjusted to accommodate logistic issues.

Four tour groups came on separate days, and each tour group was split into four small groups of 4 or 5 children and two tour leaders. The tour was led by research assistants from the second author's Pediatric Pain, Health and Communication Lab and veterinary students from the Primary Healthcare Centre. The standardized script was designed to be easily administrable, even by volunteers who do not have experience in working with young children or knowledge of pediatric pain management. Veterinary students from the Primary Healthcare Centre were trained to administer the standardized script prior to the tour by the lead researcher of the project (J. Dalley). The brief (<1 hour) structured training session included reviewing the script in detail and teaching child-friendly vocabulary for explaining medical procedures.

### 2.3. Procedure and Stimuli

The local Research Ethics Board granted approval for this study. Consent from parents and assent from children were provided immediately prior to the tour. Children were able to participate in the Teddy Bear Clinic Tour without participating in data collection. Data were collected from participating children immediately before (Time 1) and immediately after (Time 2; 45 minutes later) the tour. At both time points, the children were asked to respond to three different picture stimuli presented in a fixed order: (1) a syringe fitted with a needle (medical, negative stimulus); (2) a stethoscope (medical, neutral stimulus); and (3) a kitten (neutral, nonmedical stimulus). While it is possible that some children could have found the kitten picture to be negative in valence, the results of this study demonstrated that the children in this sample did not find this stimulus to be fear inducing. A structured script was used to introduce each picture and instruct the children in completing the response scales. Using a projector and large screen, the participants were first shown the three pictures one at a time and asked to rate their fear of each. For example, for the needle picture, participants were told* “Imagine a doctor using this needle to give you some medicine. What I would like you to think about quietly in your head is how scared you would feel if a doctor put this needle in your arm.”* The terms fear and scared were used for the following reasons: (1) young children more easily understand these terms than the term “anxiety” [26] and (2) fear is defined as involving a proximal threat to danger, and because children were asked to imagine being in a medical situation, technically we were asking participants to rate their fear rather than anxiety (for further discussion of these terms in the context of needle procedures, see [4]). Then, participants were shown each picture again and asked to rate their expected pain from the stimulus. For example, when participants were shown the needle picture again, they were told “*Imagine a doctor using this needle to give you some medicine. What I would like you to think about quietly in your head is how much hurt you would feel if a doctor put this needle in your arm.*” Finally, children were asked to report what coping strategies they would use if they were getting a needle.

### 2.4. Measures

#### 2.4.1. Fear

The Children's Fear Scale [27], a one-item scale designed for use with children, was used to collect fear ratings for each picture. The Children's Fear Scale (CFS) consists of a series of five sex-neutral faces which express an increase in fear ranging from no fear (neutral face) on the far left to extreme fear on the far right [27]. Participants responded by circling which of the five faces best represented their level of fear, with scores ranging from 0 to 4 (0 = no fear, 4 = high fear) [27]. An initial validation study for the CFS demonstrated construct validity with an alternative self-report measure of fear (*r*_*s*_ = .73, *p* < .001), as well as test-retest reliability (*r*_*s*_ = .76, *p* < .001), and interrater reliability (with parent ratings; *r*_*s*_ = .51, *p* < .001), [27].

#### 2.4.2. Expected Pain

The Faces Pain Scale-Revised [28] was used to collect expected pain ratings in response to each of the pictures. The Faces Pain Scale-Revised (FPS-R) consists of a series of six faces showing increasing levels of pain [28]. Participants responded by circling which of the six faces best represented their expected pain, with scores ranging from 0 to 10 (0 = no pain, 10 = extreme pain) [28]. The FPS-R is recommended for use with school-aged children [29], as an outcome measure in clinical trials [30], and has demonstrated high convergent validity, construct validity, and reliability [31]. The FPS-R has been used in previous studies to measure expected pain in children ages 5–12 [28, 32].

#### 2.4.3. Coping Strategies

Children were asked: “*If you were feeling scared about getting a needle, what would you do to make yourself feel better?*” A 14-item coding system (see Table 2) was constructed by the investigators using both a deductive (based on the pediatric procedural pain and distress literature [2, 12, 13, 25, 33]) and an inductive (the current data) content analytic approach to describe the variety of responses given by participants [34]. Two undergraduate research assistants independently coded all of the participants' responses. Disagreements were resolved by discussion and consensus.

## 3. Results

A total of 71 participants had parental consent and assented to participate; no data were excluded from the final analysis. As each tour group experienced the same standardized educational program, the different groups were collapsed in the analysis. All data were inputted into SPSS, version 20. Repeated measures ANOVAs were used to assess the impact of the intervention on ratings of fear and expected pain; main effects were investigated via contrasts utilizing Bonferroni corrections for multiple comparisons. Paired samples *t*-tests were used to break apart interactions found in the ANOVAs as well as comparing reported coping strategies at Time 1 and Time 2. For analyses, if assumptions were not met, corrections were applied (e.g., Greenhouse-Geisser estimates for violations of sphericity). Effect sizes are reported using Cohen's *d* and were calculated by hand [35]. The magnitudes of effect sizes are evaluated using the criteria proposed by Cohen [22, 23]. The mean ratings for fear and expected pain for all three pictures at Time 1 and Time 2 of data collection are displayed in Table 1.

### 3.1. Impact of Intervention on Participants' Reports of Fear

A 2 (Time: 1, 2) × 3 (pictures: needle, stethoscope, and kitten) repeated measures ANOVA showed no main effect of time on participants' ratings for fear, indicating that children's fear ratings did not significantly change from pre- to postintervention, *F*(1,70) = 3.12, *p* = .081. A main effect of picture was found, *F*(1.11,77.51) = 118.21, *p* < .001. Contrasts revealed that participants gave higher ratings of fear for the needle picture compared to both the kitten picture, *F*(1,70) = 111.70, *p* < .001, and Cohen's *d* = 2.37, and the stethoscope picture, *F*(1,70) = 135.14, *p* < .001, and Cohen's *d* = 2.79; both of these effect sizes are large [22, 23]. An additional contrast comparing the kitten picture to the stethoscope picture was not significant, *F*(1,70) = 1.24, *p* = .270. No significant interaction between time of data collection and medical stimuli was found, *F*(1.42,99.56) = .194, *p* = .747.

### 3.2. Impact of Intervention on Participants' Reports of Expected Pain

A second 2 (time) × 3 (pictures) repeated measures ANOVA demonstrated a main effect of time on participants' ratings for expected pain, *F*(1,70) = 7.88, *p* = .006. Specifically, mean expected pain ratings decreased significantly from Time 1 (M = 2.00, SD = 1.27) to postintervention at Time 2 (M = 1.64, SD = 1.35). A main effect of picture on participants' ratings for expected pain was also found, *F*(1.11,77.97) = 132.33, *p* < .001. Participants gave higher ratings of expected pain for the needle picture compared to both the kitten picture, *F*(1,70) = 125.90, *p* < .001, and Cohen's *d* = 2.59, and the stethoscope picture, *F*(1,70) = 149.84, *p* < .001, and Cohen's *d* = 3.35 (both are large effects [22, 23]). There was no difference between the kitten and stethoscope pictures, *F*(1,70) = 2.99, *p* = .088. Notably, the main effects found for both time and picture should be interpreted with caution given a significant interaction between time and picture. A significant interaction effect with a small effect size [22, 23] was found between time and picture type, *F*(1.37,96.19) = 15.33, *p* < .001. A significant difference with a small effect size [22, 23] was found in the expected pain ratings for the needle picture over time, *t*(70) = 4.01, *p* < .001, and Cohen's *d* = .283. However, no significant differences were found in the expected pain ratings over time for either the stethoscope picture, *t*(70) = −1.65, *p* = .103, or the Kitten Picture, *t*(70) = −.94, *p* = .351.

### 3.3. Impact of Intervention on Participants' Reports of Coping Strategies

**Figure 1:**
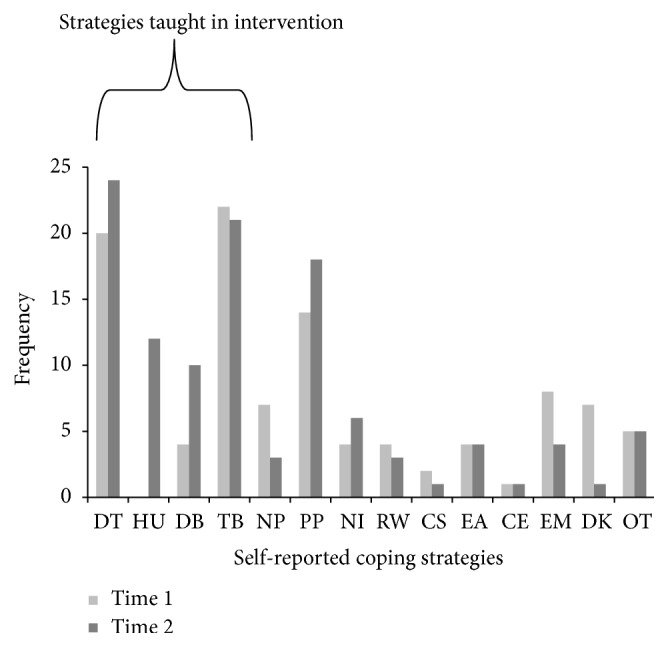
Frequencies for participants' (*n* = 71) self-reported coping strategies at Time 1 and Time 2. DT = distraction; HU = humour; DB = deep breathing; TB = use teddy bear; NP = nonphysical parental involvement; PP = physical parental involvement; NI = nonparental physical involvement; RW = reward; CS = coping statement; EA = eating; CE = close eyes; EM = emotions; DK = do not know/nothing; and OT = other. The first four strategies were taught in intervention.

Frequencies for number of strategies reported by participants at Time 1 and Time 2 were compared to determine if the intervention was efficacious in teaching participants procedural coping strategies. Please note that the categories of* emotions*,* do not know/nothing*, and* other* were not considered “valid” coping strategies and were therefore not included in the analysis of participants' reported coping strategies. Children reported a higher frequency of* intervention-taught* coping strategies at Time 2 (M = .90, SD = .72) versus Time 1 (M = .70, SD = .76), *t*(70) = −2.17, *p* = .034, and *r* = −.06, representing a significant, albeit small effect [22, 23] (Figure 1). There was no significant difference between the* total* number of coping strategies reported at Time 1 (M = 1.3, SD = .96) and Time 2 (M = 1.5, SD = .88), *t*(70) = −1.31, *p* = .194.

## 4. Discussion

When developmentally appropriate educational information is provided to children regarding medical procedures, both children and parents report decreased distress and increased satisfaction [5]. An educational program about medical procedures and procedural coping strategies could reduce children's procedural fear and pain. The objective of this study was to determine whether an educational program reduced participants' ratings of fear and expected pain from medical stimuli and increased their knowledge of procedural coping strategies.

In order to effectively help children manage fear during needles and other minor medical procedures, it is important to determine which procedures they perceive as fear inducing. Children rated the needle as more fear inducing than the stethoscope or control picture (kitten) at both times of data collection. Past research has shown that needles and needle procedures are fear inducing [4, 7, 9], and the results of this study support these conclusions as the mean rating of fear of the needle across time was in the moderate range (approximately 2 on a 0–4 rating scale). Contrary to what was hypothesized, the educational program was not successful in reducing children's ratings of fear of either of the medical stimuli. As needles were rated as moderately fear inducing by participants, perhaps a more focused educational program is needed to reduce children's fear of needles (e.g., focusing on specific aspects of the needle procedure). Additionally, as participants gave low fear ratings for the stethoscope, the failure to find reductions for that stimulus across time is likely due to floor effects; there is no literature to suggest that children are as fearful of stethoscopes as they are of needles.

An additional goal of the Teddy Bear Clinic Tour was to reduce children's ratings of expected pain from medical equipment and procedures. Participants reported expecting significantly less pain from a needle after the intervention; in fact their ratings were lower than the expected pain ratings for a group of 8–11-year-old children receiving topical anaesthetic for needle pain [36]. The decrease in ratings of expected pain for the needle after the Teddy Bear Clinic Tour is an important finding, as needles are frequent in childhood. If these pain and distress are not managed, negative outcomes such as nonadherence to immunizations (or other required needle procedures) and elevated levels of needle fear can occur [4, 7]. The current results suggest that it is possible to reduce participants' ratings of expected pain for needle procedures through a fairly brief, one-time educational program.

The Teddy Bear Clinic Tour did not significantly impact children's levels of expected pain for stethoscope procedures. Similar to the floor effects that likely impacted the results for fear ratings, the average expected pain rating for the stethoscope was 0 at Time 1. In contrast, the mean rating of expected pain across time for the needle was moderate (5 on a scale of 0–10). These findings indicate that while children may associate pain with some medical procedures and equipment, not all medical stimuli are necessarily perceived as more painful than nonmedical stimuli. Evidently, the children had already developed beliefs regarding stethoscopes and needles prior to experiencing the tour, suggesting that even young children can identify general medical equipment and understand whether that equipment is associated with procedural pain.

A central component of the Teddy Bear Clinic Tour was to provide children with coping strategies they can use during needles and other minor medical procedures to manage their pain and fear. The evidence-based coping strategies participants were taught during the tour included distraction, humour, and deep breathing [12, 13]. The Teddy Bear Clinic Tour was successful in teaching children these coping strategies, as participants reported more coping strategies taught in the educational program following the intervention; however, while statistically significant, the difference was small. It is important that children learn self-directed coping strategies as they often prefer to take an active role in their pain relief [37], which is ideal as pain management by healthcare professionals is often inadequate [7]. Therefore, teaching children these coping strategies through the educational program could potentially equip them with effective strategies to reduce pain during future medical procedures (e.g., immunizations, venipunctures).

### 4.1. Strengths, Limitations, and Suggestions for Future Research

While most information provision programs are focused on preparing children for hospitalization and surgical procedures, this pilot study was novel as it was designed to educate children about more general medical procedures, such as check-ups, immunizations, and venipunctures. Well-known measures were used to assess the impact of this creative educational program on children's fear and expected pain. Additionally, children reported the coping strategies they would use during a needle procedure. The interactive program was designed using recommendations from medical communication literature, including using developmentally appropriate language to teach children about the sensory and logistic aspects of general medical procedures. In order for the educational program to be practical, the script was easily administrable across different settings, by volunteers with a variety of vocational backgrounds. The full script of the tour included in the Appendix will allow this tour to be replicated or adapted in future research.

This preliminary study also has several limitations. Firstly, due to time constraints, this study did not include a control group. Therefore, it is possible children's postintervention expected pain ratings may have been lower due to something other than the educational program. However any history or maturation effects are highly unlikely given that Time 2 data were collected immediately after the tour. Furthermore, while it is technically possible that children's ratings may have changed due to regression to the mean effects, the initial scores were not extreme, reducing the likelihood of this issue. The design of this study addressed several threats to internal validity. For example, the recruitment and retention rate of participants was quite high, as 95% of the children participating in the Teddy Bear Clinic Tour elected to participate in data collection and all completed the study. Additionally, the instrumentation used remained consistent across data collection due to the standardized script and use of validated outcome measures. A key limitation is that children's current medical condition and children's previous medical experience were not accounted for but may impact expected and/or experienced procedural pain [14].

This was a pilot educational study that did not examine children's responses to actual medical procedures. The coping strategies taught and information provided were not in anticipation of an actual procedure but rather considered educational in nature. Children's reports of coping strategies are not always predictive of their coping behaviour [38]. Therefore, in future research, it will be important to test the effects of this intervention with an actual medical procedure in order to determine if children employed the coping strategies they were taught during medical procedures, and if they would report less fear or pain during an actual needle procedure after the educational program. Furthermore, while participants reported more intervention-taught coping strategies after the intervention, the difference was small and children were only reporting approximately one coping strategy on average. There is mixed evidence regarding the optimal time to educate children regarding medical procedures; therefore, another important component to address in future research is the timing of the intervention. Although education on the day of a procedure and ahead of time is recommended [8], if a child is extremely fearful regarding a procedure attempting preparation immediately beforehand may not be efficacious. In these cases, an educational program which is more removed from some of the threatening aspects of the situation (both in time and in setting) may be helpful.

This study was the first step in building an educational program on commonly occurring minor medical procedures. Most aspects of the tour can be replicated in almost any setting, but certain sections (e.g., physiotherapy and X-ray equipment) may need to be replaced in order for this educational program to be widely applicable. Future research could aim to tailor it for other settings, such as schools or community centres (e.g., using a “portable” tour kit that can be brought to classrooms or community centres, a tour video, or a book describing the tour).

## 5. Conclusion

This pilot study integrated best practices from the literature on medical information provision and procedural coping strategies into an educational program for children to learn about common medical procedures including check-ups and immunizations. This study demonstrated that a one-time Teddy Bear Clinic Tour focusing on medical equipment and procedural coping strategies through demonstration, modeling, and rehearsal shows some effectiveness in teaching children self-directed coping strategies and reducing their expectations of pain during needle procedures. The educational program included in this study was novel due to its evidence-based content, standardized script, and evaluation through outcome measures for fear and expected pain.

## Figures and Tables

**Table 1:** Mean fear ratings (CFS, scale of 0–4) and mean pain ratings (FPS-R, scale of 0–10) for picture stimuli at Time 1 and Time 2.

Picture		M	SD	95% CL	
				LL	UL
Needle	Fear				
	Time 1	2.24	1.63	1.86	2.62
	Time 2	2.11	1.64	1.72	2.50
	Pain				
	Time 1	5.82	3.72	4.94	6.70
	Time 2	4.47	3.82	3.56	5.37

Stethoscope	Fear				
	Time 1	.13	.38	.04	.22
	Time 2	.09	.41	−.01	.18
	Pain				
	Time 1	.00	.00	.00	.00
	Time 2	.11	.57	−.02	.25

Kitten	Fear				
	Time 1	.21	.51	.09	.33
	Time 2	.11	.36	.03	.20
	Pain				
	Time 1	.18	.57	.05	.32
	Time 2	.34	1.55	−.03	.71

**Table 2:** Coding categories for participants' self-reported coping strategies.

Graph legend	Category name	Definition	Examples
DT	Distraction (verbal, cognitive)	The child mentions thinking/talking about nonprocedural stimuli, and/or being attentive to nonprocedural stimuli *during the procedure*. This *does not* include humorous statements, statements about rewards after the procedure, or statements about eating food during the procedure.	(i) I would think about something else (ii) I would think about being done (iii) I would tell a story (iv) I would play a game

HU	Humor	The child mentions telling and/or hearing a funny story or joke.	(i) I would laugh at a joke (ii) I would tell a joke

DB	Deep breathing	The child mentions using a breathing strategy.	(i) I would take deep breaths (ii) Belly breathing

TB	Use teddy bear (physical distraction)	The child mentions bringing a stuffed animal/blanket to the procedure or holding a stuffed animal/blanket before, during, or after the needle procedure.	(i) I would hold my stuffie/blanket (ii) I would bring my stuffie/blanket

NP	Nonphysical parental involvement	The child mentions a parent's involvement in a nonphysical way.	(i) I would bring my Mom/Dad (ii) I would talk to my Mom/Dad

PP	Physical parental involvement	The child mentions a parent's physical involvement.	(i) I would hold my Mom/Dad's hand (ii) I would cuddle my Mom/Dad

NI	Nonparental physical involvement	The child mentions physical involvement from a living thing other than a parent (e.g., a doctor, sibling, or pet)	(i) I would hold my brother's hand (ii) I would pet my dog

RW	Reward	The child mentions a reward he/she will receive *after* the procedure/as a direct result of the procedure.	(i) I would get a lollipop (ii) I would get a sticker

CS	Coping statement	The child mentions a coping statement that he/she would think about during the procedure.	(i) It's not so bad (ii) Other people go through worse

EA	Eating	The child mentions that he/she would eat or drink something *during* the procedure.	(i) I would eat a snack (ii) I would drink a smoothie

CS	Close eyes	The child mentions that he/she would close his/her eyes during the procedure.	(i) I would close my eyes (ii) I would squeeze my eyes closed

EM	Emotions	The child mentions emotions or sensory experiences he/she would feel before, during, or after the needle procedure.	(i) I would feel scared (ii) I would scream in pain (iii) I would feel sad

DK	Do not know/nothing	The child reports that he/she does not know what he/she would do to make him/herself feel better or he/she would do nothing.	(i) I don't know (ii) I wouldn't do anything

OT	Other	The child gives any response that does not fit into previous categories.	(i) I would make my brother go first (ii) Sit there quietly

## Appendix

### Teddy Bear Clinic Tour Guide Script

This guide was designed to outline a tour of the Smith Lane Animal Hospital, a clinic at the Ontario Veterinary College (OVC) co-led by pairs of veterinary students and undergraduate and graduate students in psychology.

*Community Room: Introduction (Approx. 10 min)*

*Brief Welcome to the Veterinary Facility*

*“Our hospital is just like a hospital for people – we have lots of special equipment that some of you may have seen at the doctors before. You also will see some rooms and tools that will be new to you. Just like doctors for people, everything we do here is to make your pets feel better and be as healthy as they can be!”*

*Explain the Agenda for the Tour*

*“First we will split you up into groups of about 4 or 5 and then you will bring your stuffed animals to the main exam room, where the vets will teach you about different tools and give your stuffie a check-up. After visiting the main exam room, you will be taken on a tour of our hospital where you will get to see all of the different things we can offer to your stuffies and your pets! It will be a lot of fun to explore the hospital together and see all the ways vets can keep animals healthy! You will learn about how doctors keep people healthy too.”*

*Teach Coping Strategies (for All Children in One Large Group). *This is where the children will be taught two coping strategies to use throughout the rest of the tour: deep breathing and distraction. Research literature examining coping strategies for painful procedures suggests that these two strategies are the most effective, while simple reassurance (e.g., saying “it's ok” or “it's almost over”) is an ineffective strategy and should therefore not be included in this tour.

*Begin with*

*“Does anyone ever get nervous or worried about going to the doctor? Do you think your stuffie is nervous? That's ok! Everyone can get nervous, but doctors are here to help us feel happy and healthy! If your stuffie is worried about being here today, there are a few ways you can help your stuffie feel better! Can you think of any ways to make your stuffie feel better about going to the doctor?  *Praise their responses and/or connect to more appropriate answers.* One way we can help the stuffie to feel better is by talking to them about things going on outside of the doctor's office - like something the two of you like to do at playtime or the weather outside! You can also sing your stuffie a song or tell them a funny joke. Does that sound good to everyone?”*

*Belly Breathing Exercise*

*“There are certain ways that we can breathe to help us relax when we are feeling nervous – we can do something called ‘belly breathing.' It's called belly breathing because you breathe so deeply that you can feel it in your belly. I will teach you how to belly breathe so that you can show your stuffies how to do it when they are feeling scared or nervous.”*

As you provide instructions for belly breathing, try to model the steps for the children (e.g., inhale and exhale when you instruct them to do it).

*Step 1*

*“Put your hand on your belly so that you can feel when the air goes in and out of your body. While we are doing this exercise together, I want you to imagine that you have a balloon of your favourite colour in your belly! This balloon will get bigger as you breathe in, and smaller as you breathe out.”*

*Step 2*

*“Practice breathing in now. Breathe in slowly through your nose and feel your belly expand, like a balloon blowing up. Now breathe out slowly through your mouth, and feel your belly go back in, like a balloon letting out its air and flying away. Say ‘haa' as you breathe out. Try to keep your shoulders relaxed too! You are doing great so far!”*

*Note*. If any of the children seem to struggle with belly breathing, you can try suggesting different ways for them to imagine breathing. For example, you can say something like the following:
*“When you breathe out, imagine blowing all of your air down the whole length of your body, right down to your legs and feet.”*

After the children understand how to belly breathe, have them teach their stuffies.
*“Can you all show your stuffies how to take big breath slowly?* Help the kids practice taking deep breaths and praise them for their efforts.* Now that we practiced talking to our stuffies and taking deep breaths, I think we all are ready to check out some of the helpful tools doctors use to make sure our bodies are healthy.”*

Divide into small groups (of ~5 children each).

*Main Exam Room (Approx. 20 min)*. The purpose of the main exam room is to teach the children about the different instruments used by both veterinarians and doctors.

*Have the children stand on the other side of the examination table from you. Begin by introducing yourself and have all the children take turns telling you their names and the names of their stuffies. Remember to speak clearly and enthusiastically*.

1. Discuss why it is important for both people and their pets to go to the doctor.

*“We might go to the doctor if we are feeling sick, but we also need to go to the doctor – even if we feel fine – to make sure that everything is working properly and that we are strong and healthy. The same is true for your pets. Your stuffed animal is here today to have a check-up, and if anything is wrong with him/her we can help your stuffie out. After all, that's what doctors are for - to make you sure you and your stuffie are healthy and happy!”*

(2) Introduce the medical instruments.

*“Before we fix each of your stuffies, we will explain some of the tools that vets and doctors can use to look at someone's body. The doctor can't always see what's happening in your body just by looking at you, so they may need to use special tools. These tools are used to get a closer look inside someone's body to make sure everything in there is running smoothly. You and I can work together to make sure your stuffie is healthy, just like you and your doctor work together to make sure you're healthy!”*

(3) Discuss the name, function, and “feel” of needles.
Name:

*“Does anyone recognize this? This is a needle, and it can be used for a few things.”*

Function:

*“It can be used to take a little bit of your or your stuffie's blood to send for tests. The needle only takes a tiny bit of blood, and it goes into the little tube here. That's really important, because the doctor gets to look at the blood close up to make sure you and your stuffie are healthy. Needles can also be used to give medicine to the inside of your or your stuffie's body, like when you get a flu shot. We all need this medicine so that we don't get sick.”*

Feel:

*“Raise your hand if you have ever gotten a needle before. Needles are a little sharp, so when the doctors use them you will feel a pinch. Real needles have a point at the end, which means they are only safe for a doctor or trained person to use.”*

Demonstration and coping exercise:

*“Do you remember the ideas we talked about earlier that we use if we feel worried? *(Praise them for their participation if they respond.)* We can tell our stuffies to think about playtime, or we can tell them a funny story or joke! We can also show our stuffie how to take deep breaths, because this can help them feel better if they are feeling nervous. I'm going to give my teddy a needle. Can you help me to show my teddy how to take deep breaths?”*

One tour leader should give the teddy assigned to each main exam room a needle while the other tour leader helps the children practice taking deep breaths. Remember to immediately dispose of the needle and place a Band-Aid on the stuffie.

Conclude by saying the following:

*“You did a great job helping this stuffie to take deep breaths! I could tell that made this stuffie feel a lot better about getting a needle. Thanks to the needle, this stuffie got the medicine it needs so that it won't get sick!”*

After demonstrating how the needle is used on the OVC's stuffie, give each child's stuffie a “fake needle,” using a syringe without a needle in it.

(4) Discuss the name, function, and “feel” of a stethoscope.
Name:

*“Does anyone know what this is? This is called a stethoscope - can everyone say stethoscope? … Terrific!”*

Why:

*“Doctors use this to hear the different sounds inside your or your stuffie's body. Usually, the doctor can't hear sounds like your heart beat or air moving in and out of your lungs, but with the stethoscope, the sounds get much louder. It's like turning up the volume on the TV.”*

Feel:

*“Doctors might listen to your and your stuffie's heart or breathing in a couple of different places, so they might put the stethoscope on your chest or your back so that things sound louder! The metal part can feel a little chilly on your skin, but not too cold - look, I can practice on my arm.” *Put the stethoscope on different parts of your arm (or the other tour leader's) to show children it feels fine.

Demonstration:

*“Let's listen to your stuffie's heartbeat together.”  *If the children want to hear a heartbeat, put the stethoscope up to your chest (or the other tour leader's), not the children's, so they can listen.

(5) Discuss the name, function, and “feel” of an otoscope.
Name:  

*“This is called an otoscope - can everyone say otoscope? … Very good!”*

Function:  

*“An otoscope is like a little telescope with a small light on the end of it that is used to see inside of your ears better. Our ears are an important part of our body because they help us to hear people talking to us and the other sounds around us. That's why, even if we don't have an earache, it's important for doctors to look inside our stuffies and our ears to make sure they are clean!”*

Feel:  

*“It's very tiny, so you will barely feel it in your ear! However, you might hear an echo noise or a click - that just means the otoscope is working!”*

Demonstration:  

*“Let's look at the inside of your stuffie's ears together.”*

*Treatment Room (10 min)*. This section of the tour allows the children to apply the coping strategies they have learned in a needle-specific context.
*“This is where the vets get to practice using needles to take blood from animals, like dogs and cats. Remember how earlier we learned that the doctor can't always tell how healthy your stuffie is just by looking at it? And that means that your stuffie has to get a needle? Well, in this room all of the vets get to practice using needles on stuffed animals, and these animals have been filled with fake blood, kind of like something you would maybe see on Halloween. The vets practice taking blood so that when they work with a real animal they can do it quickly and try to keep it from hurting as much as possible.” *

*“Remember that needles are used for a few reasons. For both people and animals, needles can be used to take blood so that the doctor can make sure you are healthy, or they can be used to give medicine, like a flu shot.”*

*Practice Coping Exercises*

*“Can anyone tell me one of the ways that you can do to help your stuffie feel better if he/she is feeling nervous about getting a needle?” *(Praise their responses).* “Remember that there are a few things that we can do to help our stuffies during a needle.”*

- * “Think about other things, like play time, what you did that day, the weather*
- *Tell your stuffie a funny story or a joke, or read a story to them*
- *Take deep breaths to relax*
- *Play a game, or play with some bubbles. We have bubbles here in this room!”*

*Here's a Rhyme to Help You Remember*. Think about your day, a funny joke to say, bring a game to play, or take deep breaths and float away!

Allow the children to briefly practice these coping strategies with the stuffed animals in the room.

*Radiology Room (<5 min)*

*Discuss the X-Ray Machine*

*“Instead of taking a picture of the outside of your body, like a normal camera would, an X-Ray takes a picture of the bones inside your body to make sure they aren't broken and are in the right place. Having an X-ray picture taken doesn't take very long and it won't hurt you or your stuffie at all!” *

*“Raise your hand if any of you have needed to get an X-ray before.” *Praise the children for participating if any of them raise their hands.* “Okay, I will explain what it might be like for you, or for your stuffie. Only one person can get a picture taken at a time. That means that if you needed to get an X-ray picture, you would come to a room like this and your parents would have to wait outside while the doctor uses the X-ray machine with you. Some of you might feel fine about being alone with the doctor for a little bit, and even though the X-ray machine doesn't hurt at all, others might feel a little nervous. It's okay to feel nervous, but just remember that your parents would be waiting right outside for you, and that the doctors are your friends.”*

You can have the stuffed dog or cat set up with an example picture on the screen so that the children can see what an X-ray actually looks like—this is a great way to help the children's understanding.

*Discuss Broken Bones*

*“If the X-ray showed that your pet has broken one of his/her bones, the vet would put a cast on the area that your pet has injured. A cast is like a special kind of bandage that is much bigger and stronger than a normal bandage, which helps to keep the bone in place while it heals.” *

*Wet and Dry Rehabilitation Rooms (5 min)*

*Discuss the Dry Rehabilitation Room*

*“This is a rehabilitation room. Can you say rehabilitation? Great! Rehabilitation is a big word that describes different exercises we can do if our body parts are sore or haven't been used very much. The equipment in this room helps the animals to do these exercises. Doctors are experts in telling us what exercises we should do to make our sore bodies feel better, and they can help your pets and stuffies with that too! Animals need to come here to help make them feel better after surgery or speed up the recovery process.”*

*Discuss the Wet Rehabilitation Room*

*“Our pets and our stuffies might have sore joints. Does anyone know what your joints are? Your joints are places in your body where your bones meet, like your elbows or your knees. When your pets' or stuffies' joints get sore, doctors may help fix them by doing water exercises! You can see the life jackets on the walls for the dogs so that they are safe when they are doing the exercises in the water. The pool is heated and the water is gentle, which stops the dog's body from hurting and makes the exercises easier. Pets can also come to these rooms for exercise to help keep them at a healthy weight! These rooms are kind of like playrooms for pets!”*

*Community Room: Conclusion (5 min) (Everyone in Single Large Group)*

*“Thank you everyone for visiting our hospital today. We really enjoyed taking you on the tour and looking after your stuffed animals with your help. We hope that you and your stuffies had as much fun as we did today! We also hope that you learned a lot about the importance of regular check-ups, not just for your stuffies and your pets, but for yourselves as well!”*

Engage in a Quick Debrief with the Children Using Questions Such as the following:
*“What was your favourite area to visit today? What did you learn today? What can people and stuffies do to make them less nervous? *[Could review the rhyme here].* Why is it important for people and animals to go to the doctor?”*

## References

[1] Von Baeyer C. L., Spagrud L. J. (2007). Systematic review of observational (behavioural) measures of pain for children and adolescents aged 3–8 years. *Pain*.

[2] Schechter N. L., Zempsky W. T., Cohen L. L., McGrath P. J., McMurtry C. M., Bright N. S. (2007). Pain reduction during pediatric immunizations: evidence-based review and recommendations. *Pediatrics*.

[3] Albano A. M., Causey D., Carter B. D., Walker C. E., Roberts M. C. (2000). Fear and anxiety in children. *Handbook of Clinical Child Psychology*.

[4] McMurtry C. M., Pillai Riddell R., Taddio A. (2015). Far from ‘just a poke’: common painful needle procedures and the development of needle fear. *The Clinical Journal of Pain*.

[5] Jaaniste T., Hayes B., von Baeyer C. L. (2007). Providing children with information about forthcoming medical procedures: a review and synthesis. *Clinical Psychology: Science and Practice*.

[6] Jipson J. L., Melamed B. G. (2007). New approaches on the horizon: comments on Jaaniste, Hayes, and von Baeyer's ‘Providing children with information about forthcoming medical procedures: a review and synthesis’. *Clinical Psychology*.

[7] Taddio A., Chambers C. T., Halperin S. A. (2009). Inadequate pain management during routine childhood immunizations: the nerve of it. *Clinical Therapeutics*.

[8] Pillai Riddell R., Taddio A., McMurtry C. M., Shah V., Noel M., Chambers C. T. (2015). Process interventions for vaccine injections: systematic review of randomized controlled trials and quasi-randomized controlled trials. *The Clinical Journal of Pain*.

[9] Taddio A., Ipp M., Thivakaran S. (2012). Survey of the prevalence of immunization non-compliance due to needle fears in children and adults. *Vaccine*.

[10] Suls J., Wan C. K. (1989). Effects of sensory and procedural information on coping with stressful medical procedures and pain: a meta-analysis. *Journal of Consulting and Clinical Psychology*.

[11] Taddio A., Appleton M., Bortolussi R. (2010). Reducing the pain of childhood vaccination: an evidence-based clinical practice guideline. *Canadian Medical Association Journal*.

[12] Chambers C. T., Taddio A., Uman L. S., McMurtry C. M. (2009). Psychological interventions for reducing pain and distress during routine childhood immunizations: a systematic review. *Clinical Therapeutics*.

[13] Uman L. S., Birnie K. A., Noel M. (2013). Psychological interventions for needle-related procedural pain and distress in children and adolescents. *The Cochrane Database of Systematic Reviews*.

[14] Noel M., Chambers C. T., McGrath P. J., Klein R. M., Stewart S. H. (2012). The influence of children's pain memories on subsequent pain experience. *Pain*.

[15] Melamed B. G., Ridley-Johnson R. (1988). Psychological preparation of families for hospitalization. *Journal of Developmental and Behavioral Pediatrics*.

[16] Santen L., Feldman T. (1994). Teddy bear clinics: a huge community project. *The American Journal of Maternal Child Nursing*.

[17] Zimmerman P. G. (1997). Teddy says ‘hi!’: teddy bear clinics revisited. *Journal of Emergency Nursing*.

[18] Bloch Y. H., Toker A. (2008). Doctor, is my teddy bear okay? The “Teddy Bear Hospital” as a method to reduce children's fear of hospitalization. *The Israel Medical Association Journal*.

[19] Peterson L., Ridley-Johnson R., Tracy K., Mullins L. L. (1984). Developing cost-effective presurgical preparation: a comparative analysis. *Journal of Pediatric Psychology*.

[20] Klingman A. (1985). Mass inoculation in a community: the effect of primary prevention of stress reactions. *American Journal of Community Psychology*.

[21] Kajikawa N., Maeno T., Maeno T. (2014). Does a child's fear of needles decrease through a learning event with needles?. *Issues in Comprehensive Pediatric Nursing*.

[22] Cohen J. (1988). *Statistical Power Analysis for the Behavioural Sciences*.

[23] Cohen J. (1992). A power primer. *Psychological Bulletin*.

[24] Melamed B. G., Yurcheson R., Fleece E. L., Hutcherson S., Hawes R. (1978). Effects of film modeling on the reduction of anxiety related behaviors in individuals varying in level of previous experience in the stress situation. *Journal of Consulting and Clinical Psychology*.

[25] DeMore M., Cohen L. L. (2005). Distraction for pediatric immunization pain: a critical review. *Journal of Clinical Psychology in Medical Settings*.

[26] Ridgeway D., Waters E., Kuczaj S. A. (1985). Acquisition of emotion-descriptive language: receptive and productive vocabulary norms for ages 18 months to 6 years. *Developmental Psychology*.

[27] McMurtry C. M., Noel M., Chambers C. T., McGrath P. J. (2011). Children's fear during procedural pain: preliminary investigation of the children's fear scale. *Health Psychology*.

[28] Hicks C. L., von Baeyer C. L., Spafford P. A., van Korlaar I., Goodenough B. (2001). The Faces Pain Scale—Revised: toward a common metric in pediatric pain measurement. *Pain*.

[29] Stinson J. N., Kavanagh T., Yamada J., Gill N., Stevens B. (2006). Systematic review of the psychometric properties, interpretability and feasibility of self-report pain intensity measures for use in clinical trials in children and adolescents. *Pain*.

[30] McGrath P. J., Walco G. A., Turk D. C. (2008). Core outcome domains and measures for pediatric acute and chronic/recurrent pain clinical trials: PedIMMPACT recommendations. *Pain*.

[31] Tomlinson D., Von Baeyer C. L., Stinson J. N., Sung L. (2010). A systematic review of faces scales for the self-report of pain intensity in children. *Pediatrics*.

[32] Spafford P. A., Von Baeyer C. L., Hicks C. L. (2002). Expected and reported pain in children undergoing ear piercing: a randomized trial of preparation by parents. *Behaviour Research and Therapy*.

[33] Blount R. L., Corbin S. M., Sturges J. W., Wolfe V. V., Prater J. M., Denise James L. (1989). The relationship between adults' behavior and child coping and distress during BMA/LP procedures: a sequential analysis. *Behavior Therapy*.

[34] Elo S., Kyngas H. (2007). The qualitatiative content analysis process. *Journal of Advanced Nursing*.

[35] Field A. P. (2013). *Discovering Statistics Using SPSS*.

[36] Cohen L. L., Cohen R. J., Blount R. L., Schaen E. R., Zaff J. F. (1999). Comparative study of distraction versus topical anesthesia for pediatric pain management during immunizations. *Health Psychology*.

[37] Franck L. S., Sheikh A., Oulton K. (2008). What helps when it hurts: children's views on pain relief. *Child: Care, Health and Development*.

[38] Skinner E. A., Edge K., Altman J., Sherwood H. (2003). Searching for the structure of coping: a review and critique of category systems for classifying ways of coping. *Psychological Bulletin*.

